# A high-resolution large-area detector for quality assurance in radiotherapy

**DOI:** 10.1038/s41598-024-61095-2

**Published:** 2024-05-09

**Authors:** Andreia Maia Oliveira, Hylke B. Akkerman, Saverio Braccini, Albert J. J. M. van Breemen, Gerwin H. Gelinck, Natalie Heracleous, Johannes Leidner, Fabrizio Murtas, Bart Peeters, Marco Silari

**Affiliations:** 1 CERN - Occupational Health & Safety and Environmental Protection Unit, Radiation Protection Group, 1211 Geneva 23, Switzerland; 2 Laboratory for High Energy Physics (LHEP), Albert Einstein Center for Fundamental Physics (AEC), University of Bern, Sidlerstrasse 5, 3012 Bern, Switzerland; 3 Holst Centre/TNO, High Tech, Campus 31, 5656 AE Eindhoven, The Netherlands; 4 Institute of Radiation Physics, Lausanne University Hospital and Lausanne University, Lausanne, Switzerland; 5 INFN-LNF, 00044 Frascati, Italy; 6 Present Address: EBG MedAustron GmbH, Marie Curie-Straße 5, 2700 Wiener Neustadt, Austria; 7 Present Address: Medidee Services SA, Chemin de Rovéréaz 5, 1012 Lausanne, Switzerland

**Keywords:** Radiotherapy, Biomedical engineering, Electrical and electronic engineering, Applied physics

## Abstract

Hadron therapy is an advanced radiation modality for treating cancer, which currently uses protons and carbon ions. Hadrons allow for a highly conformal dose distribution to the tumour, minimising the detrimental side-effects due to radiation received by healthy tissues. Treatment with hadrons requires sub-millimetre spatial resolution and high dosimetric accuracy. This paper discusses the design, fabrication and performance tests of a detector based on Gas Electron Multipliers (GEM) coupled to a matrix of thin-film transistors (TFT), with an active area of 60 × 80 mm^2^ and 200 ppi resolution. The experimental results show that this novel detector is able to detect low-energy (40 kVp X-rays), high-energy (6 MeV) photons used in conventional radiation therapy and protons and carbon ions of clinical energies used in hadron therapy. The GEM-TFT is a compact, fully scalable, radiation-hard detector that measures secondary electrons produced by the GEMs with sub-millimetre spatial resolution and a linear response for proton currents from 18 pA to 0.7 nA. Correcting known detector defects may aid in future studies on dose uniformity, LET dependence, and different gas mixture evaluation, improving the accuracy of QA in radiotherapy.

## Introduction

Radiotherapy is a modality of cancer treatment that uses ionising radiation to kill cancer cells. It is often used to shrink or eliminate tumours, by damaging the DNA of cancer cells, which prevents them from dividing and growing. One of the main goals of radiotherapy is to deliver a high dose of radiation to the tumour while minimising the dose received by surrounding healthy tissue, in order to minimize side effects and maximize the therapeutic effect. Patient specific quality assurance (PSQA) is a crucial process in radiation therapy to verify the accuracy of treatment plans and ensure the delivery of the correct radiation dose to the intended target volume. PSQA can be conducted through either measurements or independent dose calculation systems, involving a series of tests and checks to compare the calculated dose from the treatment planning system (TPS) with the dose delivered by the treatment machine. The PSQA process typically includes comparisons of measured/evaluated and planned radiation dose distributions, aiming to identify any discrepancies between the two and address them appropriately.

The development of techniques like Intensity Modulated Radiation Therapy (IMRT) and Volumetric Modulated Arc Therapy (VMAT) allows for a better conformity of the dose to the tumour and in numerous cases offers significant improvements with respect to conventional radiotherapy^1,2^. The resulting treatment plans are often complex, involving multiple beams. The PSQA of these plans, therefore requires accurate evaluation with good spatial resolution^3^. F. S. Matar et al.^3^ emphasize the use of detectors with submillimetric precision, even employing solid-state detectors, which results in an outstandingly high spatial resolution of 0.784 mm and 0.2 mm.

Treatment with hadrons (currently protons and ^12^C ions) is a radiotherapy technique that for several types of tumours offers considerable advantages over conventional photon and electron treatments. Since its initial application in 1954, it is gaining popularity as a cancer treatment modality, with a growing number of patients benefiting from its unique properties. By the end of 2022, more than 360,000 patients have undergone particle therapy globally^4^. However, it is worth noting that despite its remarkable potential, the utilization of this cutting-edge approach remains relatively limited when compared to the conventional use of photons and electrons in radiotherapy, which annually treats around 7 million patients^5^. Hadrons are used to irradiate tumours since, unlike photons, they have the unique property of increasing energy deposition with penetration depth, with a peak at the end of the range followed by a sharp drop (Bragg curve). This characteristic provides a more conformal dose distribution to tumours, reducing the negative side-effects due to radiation delivered to healthy tissue^6^. Carbon ions have a higher relative biological effectiveness (RBE) than protons or photons i.e., the same absorbed dose leads to higher cell damage. There is considerably less dependence on biological variations between and within tumours for the response to ^12^C ion irradiation that makes this advanced modality suitable for the treatment of tumours with known resistance factors (such as hypoxia, undifferentiation and heterogeneity of the tumour) against photons and protons^7^.

To provide an effective dose delivery, radiation therapy requires instruments with high spatial resolution but also extremely accurate dose calculation. The International Commission on Radiation Units and Measurements (ICRU) states that at the level of one standard deviation, a relative accuracy of 3% is desirable, although 5% is often accepted, while a reproducibility of 2% is required^8^. The evaluation of dose and dose distribution administered to patients is a fundamental quality control process during the radiation treatment and it is even more crucial for the evaluation of the dose delivered through advanced techniques such as stereotactic radiosurgery that combines high conformity and high dose gradient. This technique has demonstrated remarkable effectiveness in treating a wide range of cancers, including brain metastases, primary non-small cell lung cancer, metastatic lung tumours, and hepatocellular carcinoma^9,10^. This form of radiation therapy delivers high doses of radiation within a few fractions and dose fall-off values can reach up to 30%/mm^11^. Additionally, a Quality Assurance (QA) procedure in hadron therapy requires not only an accurate dose calculation, but also a high spatial resolution. A high spatial resolution of the 2D dose distribution is important because most of the treatment plans in Pencil Beam Scanning (PBS) are characterized by very high “in-field” dose gradients. Distal falloff values defined as the distance between the distal position of the 80% and 20% dose levels, z80-20, at intensity-modulated proton therapy can reach 4 mm, which corresponds to a gradient of 15%/mm^12^. The positioning systems employed at particle therapy centres strive to achieve submillimetric precision. QA techniques and equipment are put in place to guarantee that these requirements are met. Detectors for monitoring important beam parameters, such as beam position and delivered dose, are essential to carry out an efficient QA procedure^13^. In routine QA checks, diverse types of dosimeters are used to measure the 2D dose distribution. An all-in-one system that offers accurate and real-time measurements with sub-millimetre spatial resolution and uniform response to the beam energy is highly desirable, but not available today.

To enhance QA capabilities and optimize treatments with hadron beams, we recently presented a first proof-of-concept based on a Gas Electron Multiplier (GEM) detector coupled to a Timepix readout for applications in radiation therapy^14^. However, the detector area was rather small (2.8 × 2.8 cm^2^) and could not be scaled up cost-effectively to cover the typical maximum field size of 20 × 20 cm^2^ in radiation therapy. In order to achieve larger areas, another prototype based on a GEM detector coupled to an optical readout was developed^12^. This detector combines a triple-GEM^15^ and a pixelated readout based on a matrix of organic photodiodes (OPDs). A more comprehensive explanation as well as the results of its characterization using X-Rays can be found in Ref.^12^. However, the obtained spatial resolution of only a few millimetres, limited by the isotropic emission of the photons, is insufficient for QA in hadron therapy (Supplementary Fig. 1). As a first attempt to improve the spatial resolution, the gap between the last GEM and the readout was reduced to minimize the impact of this effect. Even though the results with the reduced gap^16^ demonstrated an important improvement, the desired sub-millimetre spatial resolution was not achievable for a compact system with optical readout, i.e., without the introduction of collimators or lenses in the set-up^17–20^.

Based on the knowledge gained from studying the characteristics of the original detector, we developed a new prototype with the goal of enhancing the spatial resolution. This novel detector utilizes a charge readout based on a matrix of thin-film transistors (TFT) where the OPD frontplane which was present in the previous version is eliminated, leaving a TFT-only electronic readout. In this case, secondary electrons produced in the triple-GEM are guided by electric fields and are directly measured by the readout, thus avoiding the worsening of the spatial resolution by the isotropic light emission (Supplementary Fig. 1). This allows a compact, and more easily scalable device with an expected submillimetre spatial resolution fulfilling the criteria outlined in Ref.^12^.

In this article, we report on a prototype consisting of a triple-GEM detector and a 200 ppi resolution TFT-based charge readout with 60 × 80 mm^2^ active area. The novel detector can be employed in both conventional radiation therapy with X-ray photons as well as in hadron therapy using protons and carbon ions. It measures secondary electrons produced by the GEMs with sub-millimetre spatial resolution, allows reconstruction of the Bragg curve and shows linear response for proton currents from 18 pA to 0.7 nA.

## Detector design

**Figure 1:**
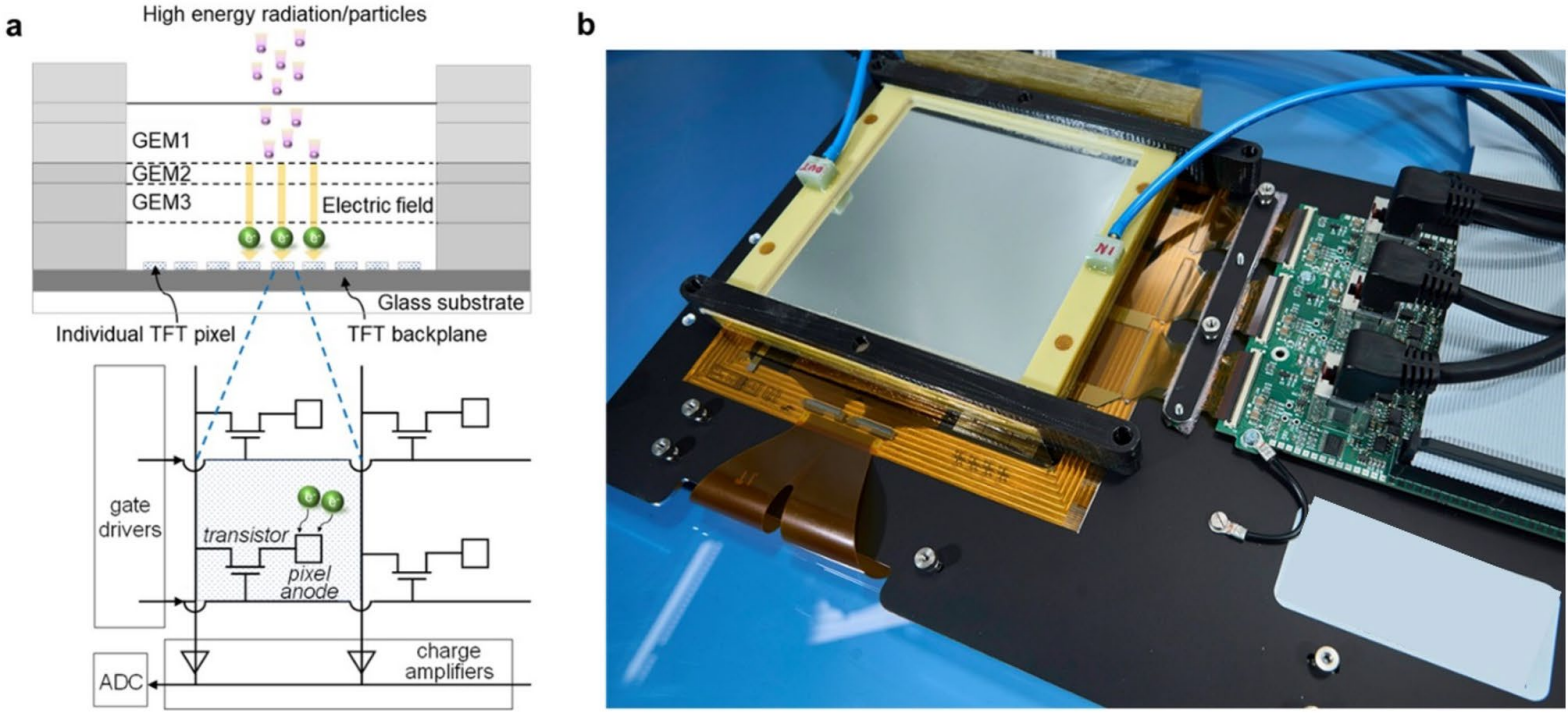
High resolution GEM-TFT radiation detector. (**a**) Schematic of the detector, consisting of a TFT backplane array and a triple-GEM frontplane. The magnification shows the TFT pixel layout, together with the drive and read-out circuit diagram. (**b**) Photograph of the detector integrated with the read-out electronics.

The new GEM-based prototype consists of a triple-GEM coupled to a pixelated readout using a TFT array. Figure 1 shows the schematic of the detector. The triple-GEM includes a 3.5 mm drift gap between the top GEM electrode (GEM1) and the cathode, which is a 15 μm thick Mylar window. The drift gap is large enough to minimize inefficiencies in charged particle detection^21^ but not too large to affect the time performance. The transfer gaps between GEM1 and GEM2 and between GEM2 and GEM3 have a thickness of 1 mm and 2 mm, respectively. The last gas region between GEM3 and the readout, identified as the induction gap, is 1 mm thick. The triple-GEM was supplied with a high voltage system (not shown in Fig. 1) designed specifically for the high voltage power supply of this kind of detector^22^. The triple-GEM detector is operated in a continuous flow of Ar/CF_4_ (90/10) or Ar/CO_2_ (70/30) gas mixture supplied at a rate of 5 l/h. These types of gas mixtures for GEMs feature attractive properties, such as high electron drift velocity and low electron diffusion^23^. Moreover, these gases are inherently safe, as they are non-flammable, which is particularly important for use in a hospital environment.

GEMs offer several advantages, including a low material budget and high amplification factors enabling single-particle detection. Furthermore, they have a well-established track record in particle detection, and their radiation hardness and aging characteristics have been documented, particularly in radiation-harsh environments like the Large Hadron Collider experiments at CERN^24^. The robust radiation resilience of GEMs, alongside the convenience of accessing standardized items, eliminates the need for custom fabrication to meet the specific requirements of this innovative detector prototype. However, drawbacks such as potential saturation at higher gains and the risk of discharges, particularly under mishandling or excessive gain conditions, should be taken into account.

GEM based detectors show a well-known exponential dependence of the effective gain on the GEM voltages. The electric fields between the top and bottom copper layers in each GEM foil can be adapted to the intensity of the beam and the type of primary particles impinging on the detector. We performed a gain scan for the different incident beams to find a stable operation condition for the detector.

The triple-GEM frontplane was coupled to a 200 ppi a- Indium Gallium Zinc oxide (IGZO) based active matrix TFT backplane. The backplane has a self-aligned dual-gate (SA-DG) TFT architecture^25,26^, which was demonstrated previously to yield a superior performance in higher current drive, steeper subthreshold slope and better determined onset voltage (V_on_) close to a gate voltage (V_g_) of 0 V. The use of a-IGZO leads to lower off-currents and off-current uniformity compared to other TFT technologies, positively impacting the noise of the readout electronics.

**Figure 2:**
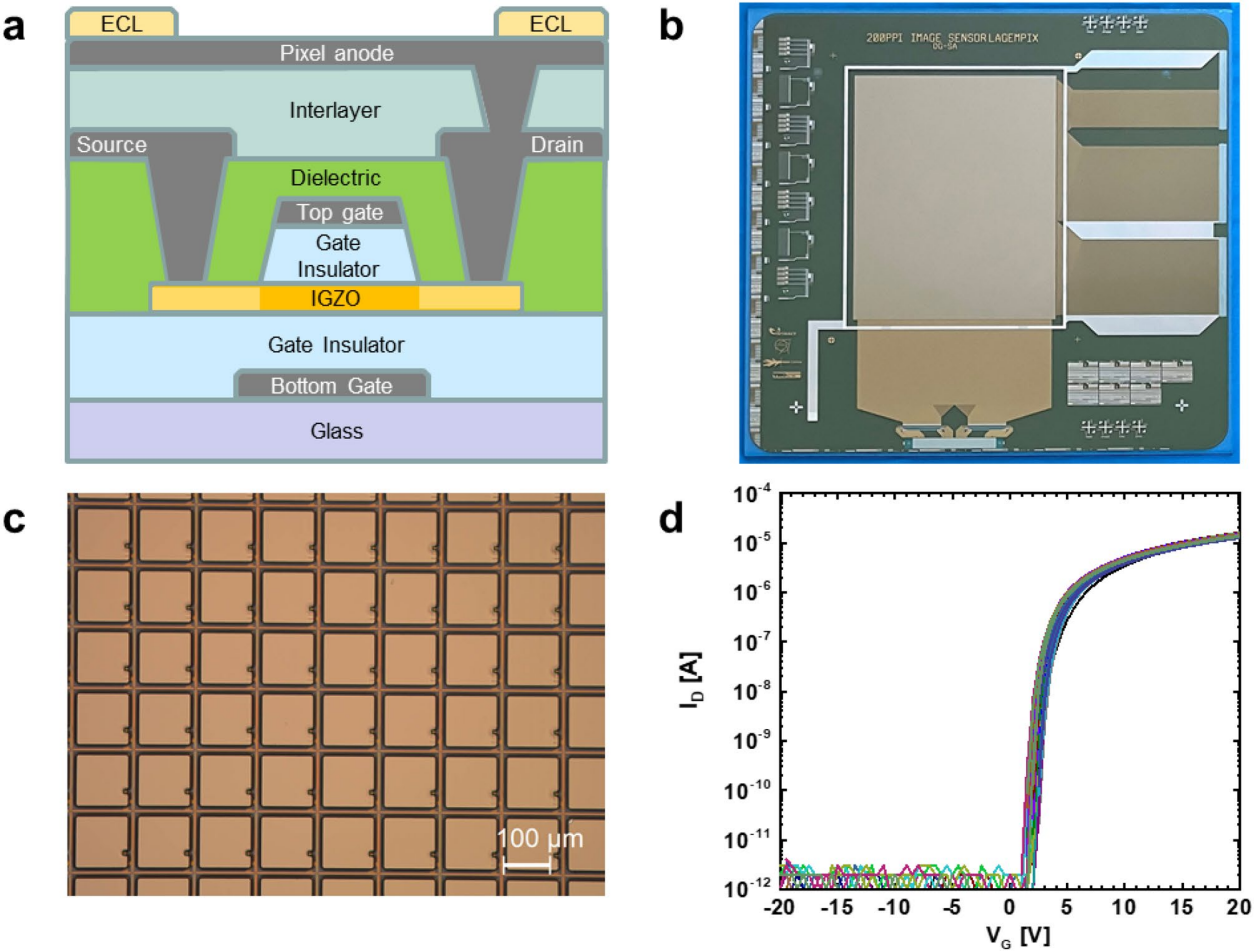
TFT backplane. (**a**) Cross section of the dual gate self-aligned TFT. (**b**) Photograph of the TFT backplane. (**c**) Microscopy image of part of the TFT backplane array showing individual pixels. (**d**) TFT characteristics of 70 dual-gate TFTs (W/L = 15/5 µm) operated in bulk accumulation mode by connecting top-gate and bottom-gate (V = V_G,bottom_ = V_G,top_).

Figure 2a shows a cross-section of the TFT backplane, monolithically integrated on a glass carrier. Connected to the TFT drain electrode is a metal contact pad (in Fig. 2a depicted by *pixel anode* in the schematic) fabricated on top of an interlayer to enlarge the effective area on which the secondary electrons from the GEM can be collected. Figure 2b and c show a photograph of the finished backplane and a zoomed image to show the individual pixels, respectively. The fabrication process of the TFT backplane consists of 6 lithographic patterning steps and is described in the Methods section. All process steps are flat panel display compatible, illustrating a facile route to mass-production. The dual-gate TFT performance is provided in Fig. 2d, where the transfer characteristics of 70 TFTs (W/L = 15/5 µm) mapped over the full 320 × 352 mm^2^ area of the motherglass are shown. The TFTs have a typical linear mobility of 29.1 ± 0.8 cm^2^/Vs, turn-on voltage (V_on_) of 0.8 ± 0.3 V, ON/OFF current ratio ∼10^7^, and a subthreshold swing of 0.4 ± 0.02 V/decade.

It is important to note that the detector—being a first prototype—exhibits several pixel defects, including non-functioning lines and other imperfections resulting from a limited yield of a repair step performed for the TFT backplane, where a design flaw was observed after completion. We would like to acknowledge that these issues are inherent to this particular backplane design and further design optimization will remove all defective lines. Tests targeting the evaluation of factors like spatial resolution were minimally impacted by the readout defects observed in the initial prototype. However, it was not feasible to perform measurements intended to assess dose uniformity due to the intrinsic nature of this early-stage prototype.

## Methods

### TFT backplane fabrication

The full stack cross-section is shown in Fig. 2a. Details of the TFT fabrication are described in Ref.^27^. As a final process step, a 1.8 µm thick SU8 edge cover / pixel definition layer is deposited and photolithographically structured to define the active pixel area.

### TFT characterization

The electrical characterization of the dual gate TFTs was performed at room temperature in a dark ambient using a semiconductor parameter analyser (4156C, Agilent). A source-drain voltage of 1 V was used in all cases. Transistor transfer characteristics were recorded by sweeping the top-gate and grounding the bottom-gate. The V_on_ of the TFTs was determined from the onset of the top-gate voltage at which the source-drain current starts to increase.

### Drivers and read-out

The gate drivers (Sitronix ST5041) and readout integrated circuits (Analog Devices AD71124) are bonded to the fan-out electrodes. The detector response was measured using a custom-made electronic system and read-out software (LabVIEW based). The detector is biased using a custom-made board and connected to an FPGA digital interface that reads the data and connects to the computer.

The readout was set to the sensitivity level of 0.5 pC, so that the least significant bit (LSB) of the 16-bit readout corresponds to a charge of approximately 47 electrons. The maximum frame rate is 95 fps (22 µs line time × 480 lines). The standard frame rate used for the majority of the experiments was 1 fps. However, during the clinical evaluation with photons utilizing the commercial Linac TrueBeam Varian, a frame rate of 10 fps was employed. The sum of all pixels for the selected ROI for 200 recorded images was averaged. The LSB value measured by the TFT-based readout vs high voltage gain follows an exponential trend as expected with low-energy (40 kVp X-rays) and high-energy (6 MeV) photons, protons and carbon ions of clinical energies.

### GEM-TFT detector characterization

The GEM-TFT detector was characterized using low energy X-rays (30–40 kV) at the Calibration Laboratory of CERN Radiation Protection Group^28^. To evaluate the spatial resolution, we placed an X-ray test pattern in front of the Mylar window to calculate the modulation transfer function (MTF). The MTF measurements were performed according to a method previously described^12^.

Next, the detector was irradiated with 17.5 MeV protons at beam intensities of up to 1.3 nA using the research Beam Transfer Line (BTL) of the IBA Cyclone 18 MeV cyclotron in operation at the Bern University Hospital (Inselspital), which is used as an irradiation facility for multi-disciplinary research^29,30^. In order to measure and control the beam characteristics during the irradiations, a UniBEaM detector^31^ was installed in the BTL, as shown in Supplementary Fig. 2a. The UniBEaM measures the beam profiles in the *x* and *y* transverse directions by passing scintillating fibres through the beam. The protons are extracted into air through a 50 μm stainless steel window facing the Mylar window. The parameters of the cyclotron operation were selected aiming for a uniform dose to the 3 cm diameter target, which is the diameter of the beam exit window.

Discrete dual-gate TFTs (channel width 15 µm, channel length 5 µm), were irradiated with three different doses (0.5, 28, 250 kGy) to evaluate their radiation hardness. A strip with multiple TFTs was placed perpendicular to the proton beam, as shown in Supplementary Fig. 2b. Protons directly hit the TFT with the active area facing the exit window (Supplementary Fig. 2c), which is the same orientation in which the TFT backplane was coupled to the triple-GEM detector (Fig. 1a and Supplementary Fig. 2a). An aluminium disk was placed behind the sample, connected to a Keysight B2985A electrometer to measure the beam current simultaneously. All electrodes were kept floating during the irradiation.

### Clinical evaluation with photon and proton/carbon ion beams

We tested the GEM-TFT detector flushed with ArCO_2_ (70:30) using a commercial Linac TrueBeam Varian using a 6 MV flattening filter (FF) photon beam at Zentrum für Strahlentherapie in Freiburg (Supplementary Fig. 3). The depth dose curve measured for the 6 MV photon beam has two distinct regions: the characteristic build-up region close to the surface and the charge particle equilibrium region at deeper depths^32^. To avoid perturbations in the measurements as a result of the contamination of electrons in the build-up region, solid water slabs with a total thickness of 1.7 cm were positioned in front of the detector. The detector was centred visually in the luminous field with the help of the lasers. The detector was inserted in the vertical support and perpendicular to the radiation beam (Supplementary Fig. 3).

The GEM-TFT was finally tested at CNAO (*Centro Nazionale di Adroterapia Oncologica*, the Italian National Centre of Oncological Hadrontherapy sited in Pavia) with the beams used in cancer treatment. The CNAO synchrotron provides proton beams with kinetic energies from 60 to 250 MeV and carbon ion beams in the range 120–400 MeV/u^33^. The beam intensity for protons varies from 10^9^ to 10^10^ particles/s, while for carbon ions it ranges from 4 × 10^7^ to 4 × 10^8^ particles/s. We performed two types of measurements with two different set-ups.

Firstly, an integrated system consisting of a commercial water phantom and the GEM-TFT was used to perform depth scans (Supplementary Fig. 4a). A light-tight and waterproof box with a wall thickness of 10 mm was designed and manufactured to house the GEM-TFT detector in water. The box is made of black polymethyl methacrylate (PMMA, type: PLEXIGLAS NOIR 811—PERSPEX 9T30). Additionally, two supports with rails to guide and fix the baseplate inside the box were designed and manufactured.

Secondly, the detector was placed directly on the treatment couch (Supplementary Fig. 4b). The detector was positioned perpendicular to the beam in a vertical custom-made structure providing a well-aligned set-up. To evaluate the spatial resolution using the edge response method, we placed a high-Z material of variable thickness in front of the detector covering a portion of its active area to create a sharp edge as shown in Supplementary Fig. 4b.

## Results and discussion

### Characterization of the GEM-TFT detector

To find the correct working point (voltages) that should be applied to the triple-GEM, it is necessary to perform a gain scan. The response obtained by summing all pixel values versus the sum of the GEM gas amplification voltages shows the expected exponential dependence. This was the expected behaviour since the gain, i.e., the ratio of the number of electrons produced over the number of primary electrons, depends exponentially on the applied GEM voltages^15^. Besides that, a current scan using photons at beam intensities ranging from 5 mA up to the maximum current of 25 mA available in the X-Ray generator was performed. The GEM-TFT detector demonstrates a linear dose response across the entire range (R^2^ = 0.9965).

**Figure 3:**
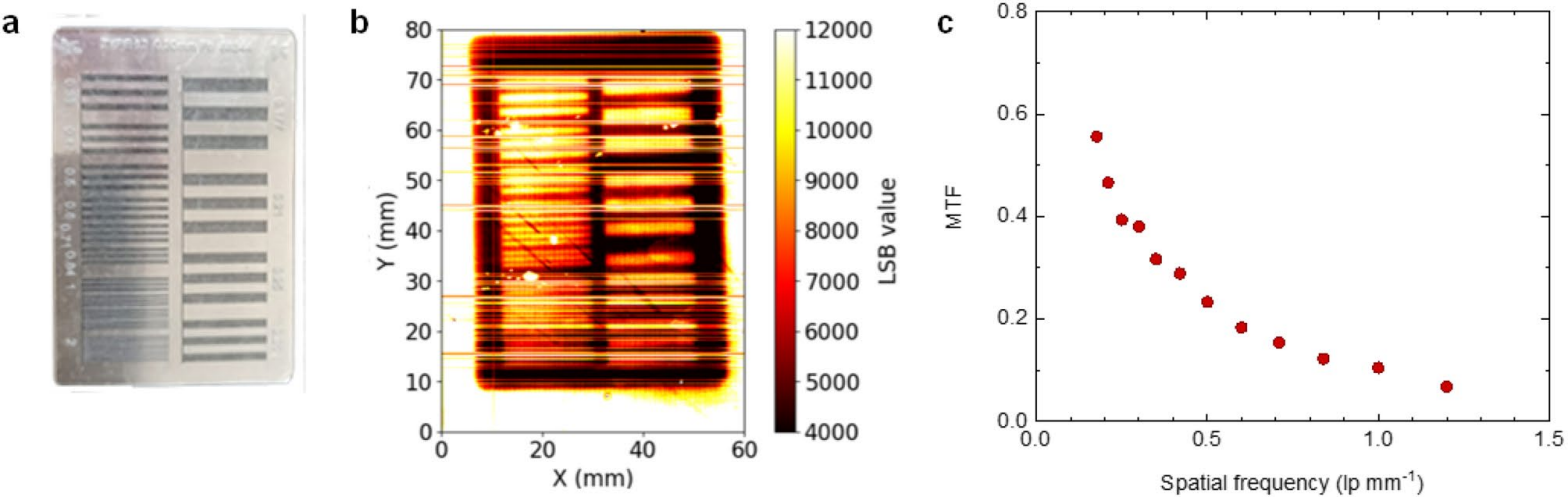
Spatial resolution. (**a**) Line pair mask type 17 made of 0.02 mm thick lead used as an imaging target. (**b**) Heat map of the TFT readout after irradiation with 30 kV X-rays. (**c**) Measured MTF with mask type 17 for the GEM-TFT detector.

The modulation transfer function (MTF) was acquired using a lead mask with a bar pattern, a common method to determine the spatial resolution^34^ and identical to the procedure used for our previous optical detector^12^. These tests were performed using 30 kV X-rays with the N-5 filter to increase the contrast of the output image. The lead mask is 0.2 mm thick with a resolution of 0.177 to 3.33 LP/mm, as shown in Fig. 3^35^. The detector yielded an MTF below 10% at 1.2 LP/mm, which corresponds to 0.83 mm spatial resolution. This sub-millimetre resolution confirms the state-of-the-art performance when benchmarked against the 2.5 mm resolution of the commercial OCTAVIUS^®^ 1000 SRS detector^36^.

**Figure 4:**
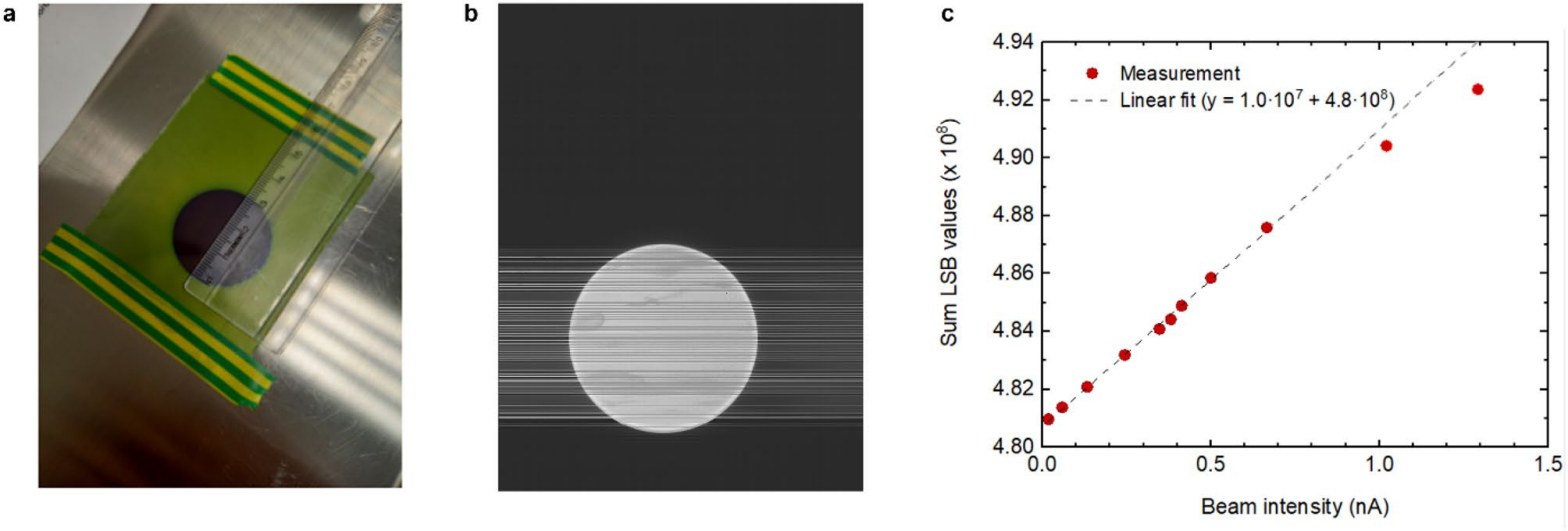
First functional test using protons at the Bern medical cyclotron. (**a**) Acquired image showing the circular shape of the beam using a GAFCHROMIC® EBT3 film. (**b**) Acquired image showing the circular shape of the beam using the GEM-TFT detector. (**c**) LSB value vs beam intensity of the detector using the 18 MeV cyclotron. The dashed line represents the linear fit to the data up to 0.7nA ().

The first experimental test at the Bern cyclotron using protons with the GEM-TFT system is shown in Fig. 4b. The protons are extracted into air by means of a 50 μm stainless steel window with a circular shape. A GAFCHROMIC® EBT3 film was placed in front of the exit window to inspect the shape of the beam. The irradiated film indicates a 3 cm diameter circle as shown in Fig. 4a. The measured result with the GEM-TFT detector showed that the full width at half maximum (FWHM) for the beam was 3.024 ± 0.005 cm. The horizontal non-functional lines in the readout images in Figs. 3b and 4b are line defects in the TFT array. These measurements confirm that is possible to study the beam size and shape indicating that the prototype is a promising tool for beam diagnosis. Additionally, a current scan with 18 MeV protons at beam intensities up to 1.3 nA was performed. For a uniform beam of 3 cm diameter, 1.3 nA corresponds to a beam intensity of approximately 10% of the maximum beam intensity for protons used at CNAO^33^. We measured a linear behaviour between 18 pA and 0.7 nA with the sum of the GEM voltages equal to 600 V as shown in Fig. 4c. Nevertheless, the two data points acquired for currents exceeding 1 nA exhibit unexplained non-linearity. To gain a better understanding, further investigations are planned.

Radiation hardness plays an important role in devices, which may be subjected to 100 Gy weekly radiation dose in a routinely operated particle therapy centre. To evaluate the radiation hardness of the prototype, we compared the background in the absence of the beam and the response to a uniform 40 kVp X-Ray field before and after a uniform irradiation with protons.

**Figure 5:**
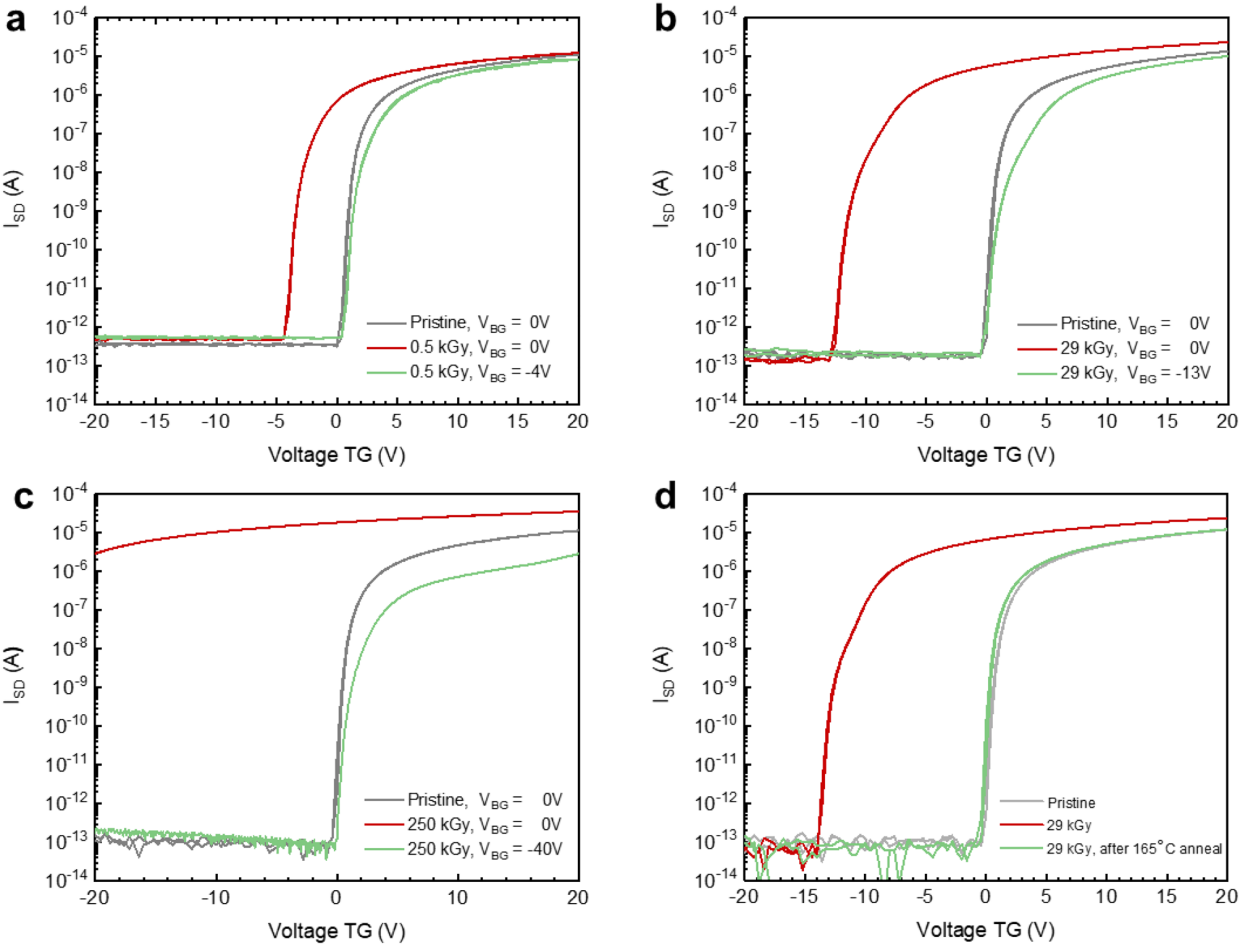
Radiation hardness of dual gate TFTs. (**a**–**c**) Id–Vg transfer characteristics before (grey), after irradiation (red) with 0.5, 29 and 250 kGy 18 MeV protons respectively, and after compensation of the V_on_ shift (green) by applying a bottom gate bias of − 4, − 13 and − 40 V respectively. (**d**) Id–Vg transfer characteristics before (grey), after irradiation (red) with 29 kGy 18 MeV protons, and after recovery by a thermal annealing process for 1 h at 165 °C (green).

Figure 5 shows a comprehensive overview of the radiation hardness results. The grey curves correspond to the transfer characteristics of TFTs in their pristine state, before irradiation. V_on_ is close to 0 V in all cases. After exposure to 0.5, 28, 250 kGy of 18 MeV protons, V_on_ shifts to − 4 V, − 13 V and ~  − 40 V respectively (Fig. 5a–c, red curves). Such a negative V_on_ shift is typically observed for oxide based TFTs when exposed to high energy radiation (X-rays^37,38^ and 5 MeV protons^39^) and attributed to an increase in electron concentration of the a-IGZO TFT active layer. The shift in V_on_ can however be compensated for in our dual-gate TFT technology. From the green curves in Fig. 5 it can be observed that by negatively biasing the bottom gate, the V_on_ of the TFTs can be clearly tuned over a large voltage range and shifted back to its initial state at about 0 V. The radiation induced V_on_ shift has a temporal character. With time, the device characteristics return to their original position. The recovery process of the TFT can be accelerated by thermal annealing. Figure 5d shows the fully recovered V_on_ to its initial state after an annealing process for 1 h at 165°C.

A detector used for treatment plan verifications in a busy hadron therapy centre is exposed to a dose of approximately 100 Gy per week. This means that the dose of 29 kGy received by the second TFT sample over a short period of time would be equivalent to approximately 6 years of operation of a commercial detector. The relatively small shift at relevant radiation doses combined with its temporal character led us to believe that the proton radiation hardness of our IGZO TFTs will be sufficient for first applications.

### Clinical evaluation with photon and proton/carbon ion beams

**Figure 6:**
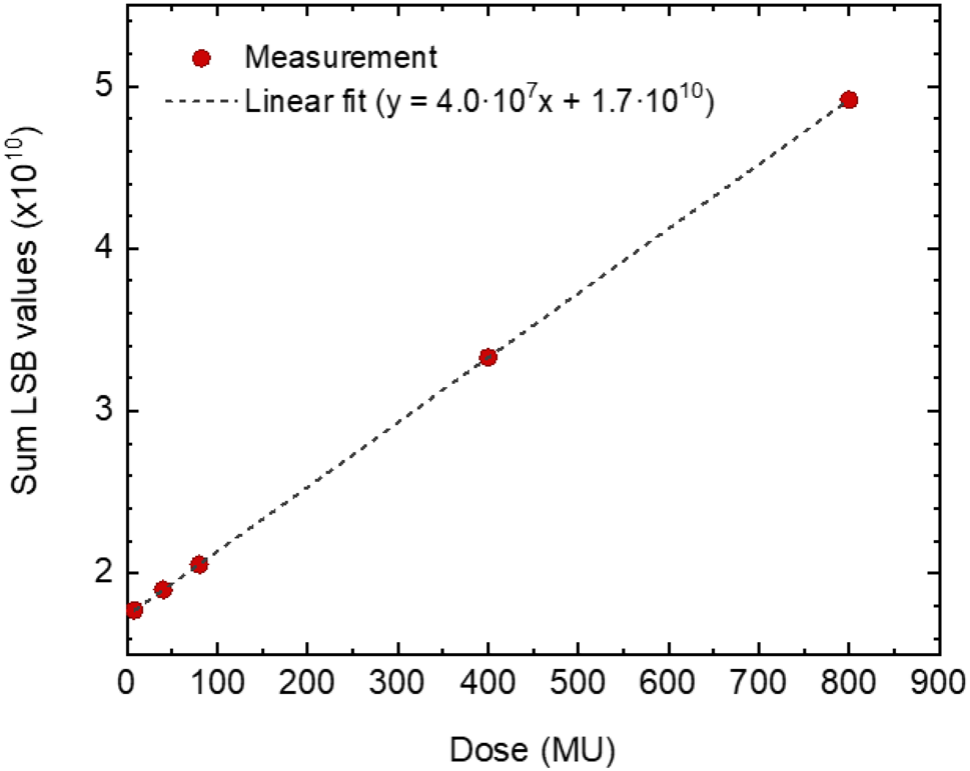
LSB value vs dose expressed in MU of the detector using the commercial Linac TrueBeam Varian. The dashed line represents the linear fit to the data.

The electron linear accelerator was calibrated to administer a dose of 100 Monitor Units (MU) for a dose rate dependence from 100 MU/min to 600 MU/min, approximately 0.8 Gy, for a 10 × 8 cm^2^ field. The dose rate at 6 MV FF beam can reach 600 MU/min. After a gain scan, the detector was operated with the sum of the GEM voltages equal to 750 V. The readout was set to the highest sensitivity level of 0.5 pC and the frame rate was 10 fps. A relative difference of 4.4% between the response to the lowest (100 MU/min) and the highest dose rate (600 MU/min) was observed. We performed a dose scan with 6 MV photons and we measured a linear behaviour between 8 and 800 MU as shown in Fig. 6. The highest dose rate available of 600 MU/min, approximately 6 Gy/min, was selected.

Electronic portal imaging devices (EPID) dosimetry, also referred to as Portal dosimetry, presents notable advantages for patient-specific quality assurance (PSQA) in IMRT and VMAT^40,41^. EPID systems offer exceptional spatial resolution and sensitivity, which is crucial for precise dosimetry measurements and PSQA tasks. Study^40^ reports a-Si 1000 flat panel imager from Varian Medical Systems featuring a phosphor screen, 1.0 mm Cu build-up layer, and a hydrogenated a-Si:H photodiode array with a 30 × 40 cm^2^ detection area with 768 × 1024 pixels, achieving a spatial resolution of 65 pixels per inch (PPI). The spatial resolution obtained in this study with the GEM-TFT detector (pixel size of 0.126 mm) is 200 PPI, hence a factor of three higher as compared to the EPID.

The process of converting pixel values to dose in Gray at the reconstruction plane and its calibration procedure are complex^42,43^. For Elekta Synergy machines a physics model was established based on manufacturer recommendations to translate EPID panel pixel data into dose through a four-step approach^41^. Absolute dosimetry with the GEM-TFT requires a comparable calibration procedure as well. Nevertheless, they stand out as sensitive and accurate dosimetry tools, requiring relatively short setup times and boasting user-friendly operation. This characteristic makes the EPID panel a more practical choice for dosimetry compared to other detectors like the full characterized GEM-TFT detector that demand a longer setup procedure.

During the first measurements with the GEM-TFT in a water phantom at CNAO using protons and carbon ions, reference measurements of the beam intensity were performed with the Dose Delivery System (DDS) of CNAO. The CNAO DDS includes the beam monitors and the control unit that constitutes the data acquisition system. It is a CE-marked medical device in clinical use at both CNAO and MedAustron EBG^33,44^. The GEM-TFT detector response shows a good agreement with the CNAO DDS (Supplementary Fig. 5).

An accurate measurement of the Bragg peak is important because it allows for precise targeting of the tumour. The Bragg curves measured with the GEM-TFT were compared to the Peakfinder data acquired with equal beam parameters. The PTW Peakfinder (PTW, Freiburg, Germany) is a commercial device for measuring the depth dose profile of a particle beam in water, containing a height adjustable water column and two large-area ionisation chambers.

**Figure 7:**
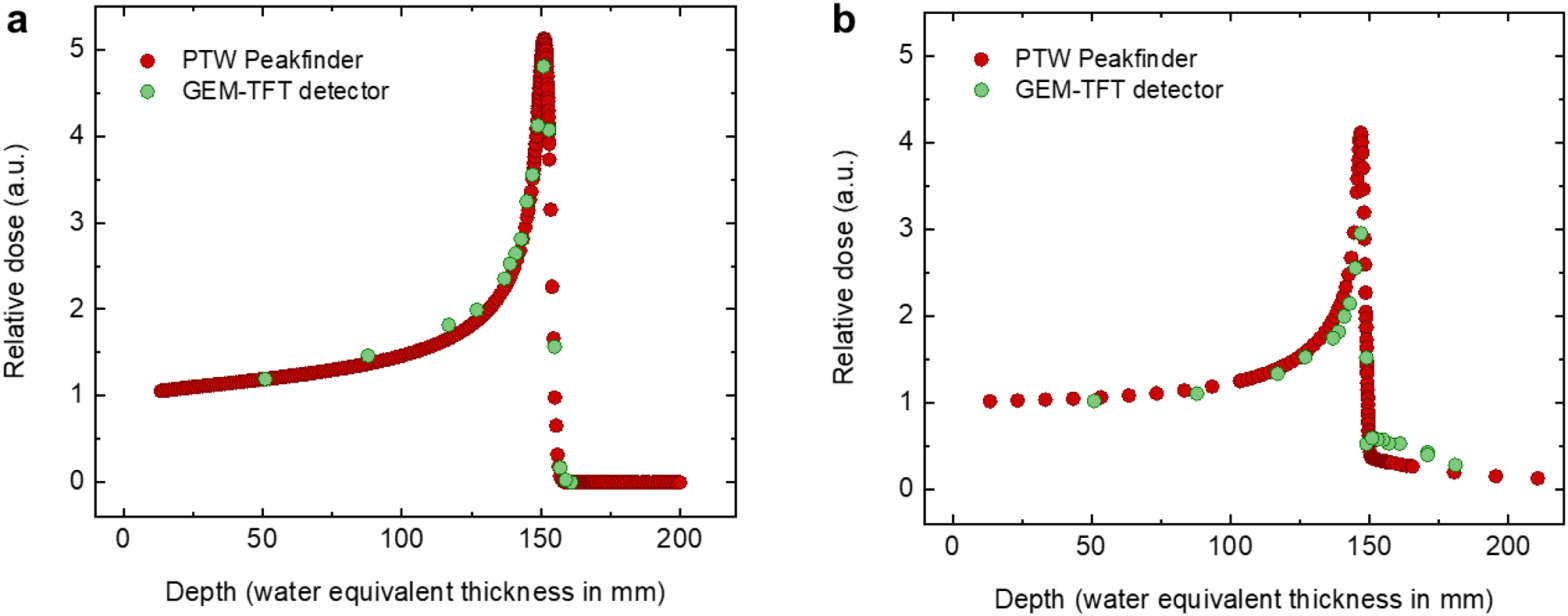
Bragg curves. Increasing energy deposition with penetration depth, with a maximum at the end of the range (151 mm) followed by a sharp decrease. The curves are normalized to unity in the plateau region (50 mm depth in water). (**a**) Bragg curve measured with the GEM + TFT () and PTW Peakfinder () with 147.72 MeV protons. (**b**) Bragg curve measured with the GEM + TFT () and PTW Peakfinder () with 207.97 MeV/n carbon ions.

Figure 7a shows the Bragg curve measured with the GEM-TFT with 147.72 MeV with an approximate 13 mm spot size, the clinical beam intensity was set at 2 × 10^9^ particles/s. Additionally, Fig. 7b shows the Bragg curve with 207.97 MeV/n carbon ions with a 6.5 mm spot size, the clinical beam intensity was adjusted to 10^7^ particles/s. The Bragg peak is evident at a depth of around 150 mm and the tail beyond the Bragg peak produced by the carbon fragments is well visible.

The GEM-TFT detector and the PTW Peakfinder present similar results for the Bragg curve of protons. Excluding the tail points, the maximum difference of 9.9% was measured at 116.9 mm. The comparison for the Bragg curves of carbon ions yields a larger difference between devices. There is a discrepancy in the maximum of the peak where the value measured with the prototype is 16.7% lower than expected. In contrast, in the tail the measured values were higher.

No saturation effect was observed with protons or carbon ions for any depth with the selected settings. The reason for the observed results with carbon ions remains unclear. One possible explanation could be a dependence on linear energy transfer (LET).

Additionally, the discrepancy could be attributed to the larger surface area of the GEM-TFT compared to the ionizing chamber of the PTW Peakfinder. This difference potentially results in a greater collection of secondary particles, subsequently augmenting the signal beyond the Bragg peak. The area can exert a significant influence. The Peakfinder has a diameter of 57.6 mm and an area of 26 cm^2^. In contrast, the GEMs used in this prototype measure 10 × 10 cm^2^ (ratio 3.8), while our readout—the relevant parameter in this context, as it is smaller than the sensitive area—encompasses 6 × 8 = 48 cm^2^ (ratio 1.8). The entrance window of the measuring chamber of the PTW Peakfinder is 0.0702 ± 0.0084 cm. In the absence of the measured value, we employed a simple SRIM-2008 simulation^45^ to estimate the water-equivalent thickness of our prototype. Specifically, our assessment pertained to a 116 MeV/n carbon ion beam in water in comparison to the GEM-TFT detector inside the water phantom. The discernible variance in range when employing the detector was 0.0442 cm. No relevant impact on the beam fragmentation is expected. Nevertheless, it is worth noting that the distinct materials involved in the GEM chamber and in the readout can influence the characteristics of the Bragg curve. To illustrate this phenomenon, we have selected two pertinent studies that effectively highlight the impact of different materials. A study^46^ was conducted to investigate the water-to-detector stopping power ratio for the plateau region. Additionally, the influence of the quartz window on the Peakfinder was investigated through comprehensive studies conducted at Heidelberg^47^. Moreover, alterations in the detector's response due to uneven distribution of defects might lead to significant signal variations. Further tests with an improved version of this prototype with no production defects could help to improve these measurements.

**Figure 8:**
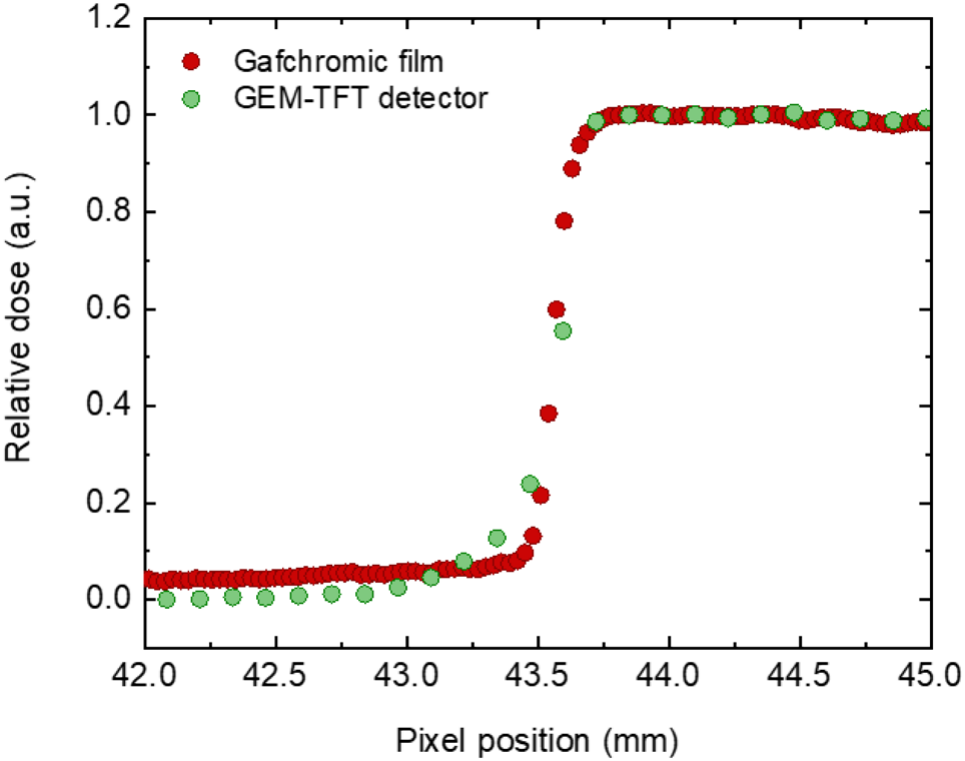
Spatial resolution test under clinically relevant hadron therapy settings. Edge response profile for 147.72 MeV protons using high Z material measured with the GEM-TFT detector () and GAFCHROMIC^®^ EBT3 films ().

Finally, the spatial resolution of the GEM-TFT detector was determined under clinically relevant hadron therapy settings using the experimental set-up shown in Supplementary Fig. 4b. Figure 8 shows a comparison of the detector response with GAFCHROMIC^®^ EBT3 films^48^, the gold standard for this type of measurements in radiation therapy. Employing in-room lasers, we achieved millimetric precision in the alignment of the collimators in relation to the detector. While quantifying the potential impact of misalignment and scattered ions on the wedge face proves complex due to multiple influencing factors, such as beam energy and material properties, the inclusion of films in this experiment facilitates a thorough evaluation. This approach effectively discerns our GEM-TFT detector's performance attributes from external variables. The FWHM obtained by the edge response method for different lines was consistently below 0.50 ± 0.05 mm, corresponding to a resolution of 1 lp/mm. The sub-millimetre spatial resolution obtained using X-rays was nicely confirmed in the hadron therapy setting. A slight difference in the response of the detector compared to that of the Gafchromic film can be seen within the range of 43.0 to 43.5 mm.

## Conclusions

In this paper we presented for the first time the applicability of flat panel TFT technology as GEM detector readout to obtain large area beam imaging with high spatial resolution at high frame rates. The goal was to explore the feasibility of this promising technology which offers benefits from larger scale production in semiconductor industry. We can see potential improvements such as combining advantages of a gaseous detector (low material budget, potentially tissue-equivalent gases) with high-resolution readouts, where ICs have limitations in terms of pixel size/number of detectors for a matrix. The sub-millimetre spatial resolution is achieved by the direct readout of the charge, the directionality of the charges from the GEM and the high pixel density of the TFT array. Moreover, the effective resolution is bolstered by the short 1 mm distance between the last GEM and the readout. We demonstrated that the detector can measure secondary electrons produced by the triple-GEM structure under high intensity beams of various types and sources of high-energy radiation, i.e., X-rays, protons, and carbon ion beams. Dose measurements conducted with 6 MV photons displayed linear behaviour within the range of 8 MU to 800 MU. Similarly, dose-rate measurements using protons, with an intensity range spanning two orders of magnitude demonstrated a linear response. Moreover, the detector showed relatively low radiation damage, that can be compensated by virtue of the two separate gate electrodes in the TFT and maximum dose difference to the reference detector of up to 10%. The observed LET dependence seems to have reduced prominence compared to well-known quenching detectors like plastic scintillators; however, further comprehensive investigation is necessary to study this phenomenon and confirm this observation. We therefore believe that this type of detector has potential for quality assurance in cancer radiation therapy.

Engineering challenges remain and have a potentially large impact on feasibility and costs, but solutions seem at least possible. The presence of line defects in the GEM-TFT detector limited our ability to assess dose uniformity using the current detector. We understand the importance of dose uniformity measurements and plan to investigate this further in future studies, utilizing advancements in both design and fabrication of TFT readout technology. The future development of a 200 × 200 mm^2^ prototype with a high-quality readout and reduced defect level, would allow for further testing of the technology in view of its potential commercialization. Since IGZO based TFT technology is nowadays used in liquid–crystal display (LCDs), organic light-emitting diode (OLED) displays and X-ray detectors at different panel sizes and resolution, we foresee a clear roadmap for the commercial use of this readout technology in GEM based large-area detectors for quality assurance in radiotherapy. Ensuring gas tightness is a crucial step in successfully creating a commercial product suitable for hospital settings. An option with a sealed or recirculating gas system can eliminate the need for gas bottles in a clinical environment, simplifying the detector operation.

### Supplementary Information

Supplementary Figures.

## Data Availability

The datasets generated and analysed during the current study are available from the corresponding author on request.
