# Supplementary material for: A high-resolution large-area detector for quality assurance in radiotherapy
Source: Sci Rep. 2024 May 9;14:10637. doi: 10.1038/s41598-024-61095-2 (PMC11082155; doi:10.1038/s41598-024-61095-2)
Supplement: Supplementary file 1 — Supplementary Figures. [file 41598_2024_61095_MOESM1_ESM.pdf]

Supplementary information

A high-resolution large-area detector for quality assurance in radiotherapy

Andreia Maia Oliveira[1,2,3*], Hylke B. Akkerman[4], Saverio Braccini[2], Albert J.J.M. van Breemen[4], Gerwin H. Gelinck[4], Natalie Heracleous[1,5], Johannes Leidner[1,6], Fabrizio Murtas[1,7,†], Bart Peeters[4], Marco Silari[1]

[1] CERN - Occupational Health & Safety and Environmental Protection Unit, Radiation Protection Group, 1211 Geneva 23, Switzerland

[2] Laboratory for High Energy Physics (LHEP), Albert Einstein Center for Fundamental Physics (AEC), University of Bern, Sidlerstrasse 5, 3012, Bern, Switzerland

[3] Now at EBG MedAustron GmbH, Marie Curie-Straße 5, 2700 Wiener Neustadt, Austria

[4] Holst Centre/TNO, High Tech Campus 31, 5656 AE Eindhoven, The Netherlands

[5] Institute of Radiation Physics, Lausanne University Hospital and Lausanne University, Lausanne, Switzerland

[6] Now at Medidee Services SA, Chemin de Rovéréaz 5, 1012 Lausanne

[7] INFN-LNF, 00044 Frascati, Italy

[†] Deceased August 2022

[*]andreia.cristina.maia.oliveira@cern.ch

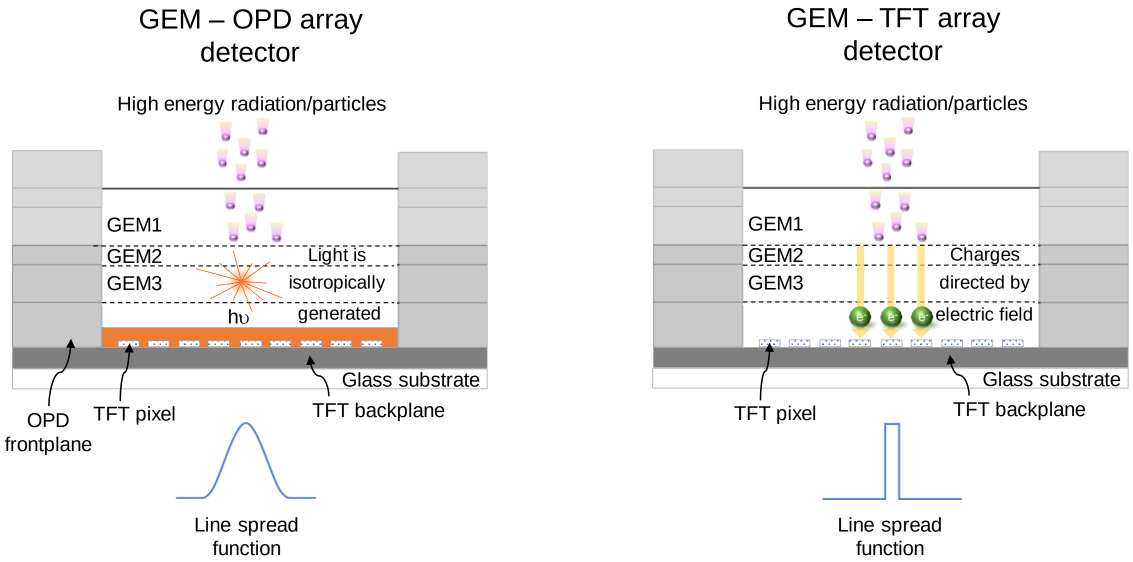

Supplementary Fig. 1 | Two configurations of GEM-based radiation detectors. a, Schematic of the GEM – OPD array detector, consisting of a TFT backplane array, an OPD frontplane and a triple-GEM frontplane. **b,** Schematic of the GEM – TFT array detector, consisting of a TFT backplane array and a triple-GEM frontplane.

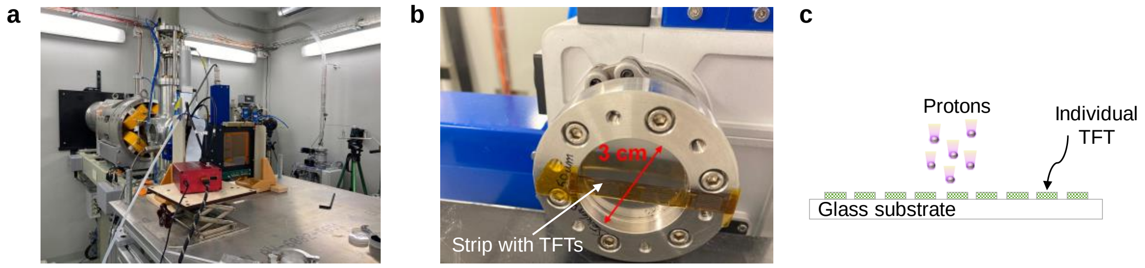

Supplementary Fig. 2 | The setup used for the proton beam tests at the BTL of the Bern medical cyclotron. a, The GEM-TFT detector and the UniBEaM detector. **b,** The exit window with an aperture of 3 cm in diameter and one PEM (white arrow) for the radiation hardness tests. **c,** Schematic view of strip of TFTs directly irradiated by the proton beam.

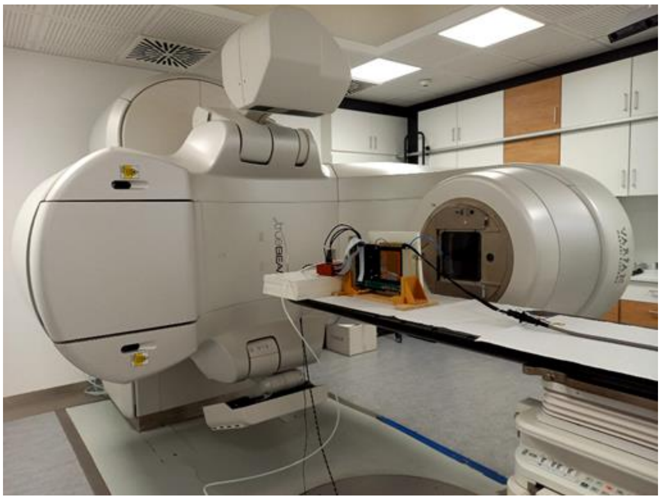

Supplementary Fig. 3 | Setup used for the photon beam tests with the Linac TrueBeam Varian. The GEM-TFT detector was placed on the top of the treatment couch and the treatment head was placed at 90.

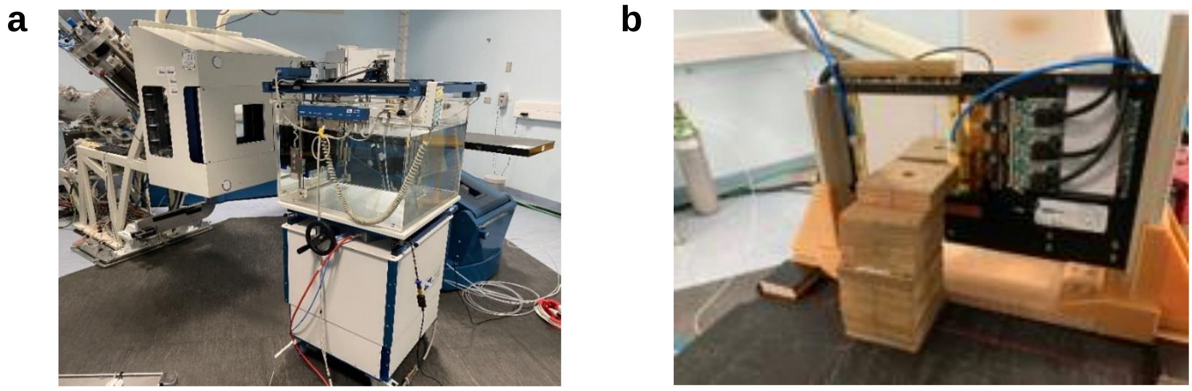

Supplementary Fig. 4 | Setup used for the proton and carbon ions beam tests at CNAO. a, The GEM+TFT detector is housed in a waterproof PMMA box attached to the water phantom's positioning system. The distance between the entrance window and the PMMA box can be altered along the beam axis. **b,** The detector was inserted in the vertical support and placed on the treatment couch, with high-Z material covering a portion of its active area to study the spatial resolution.

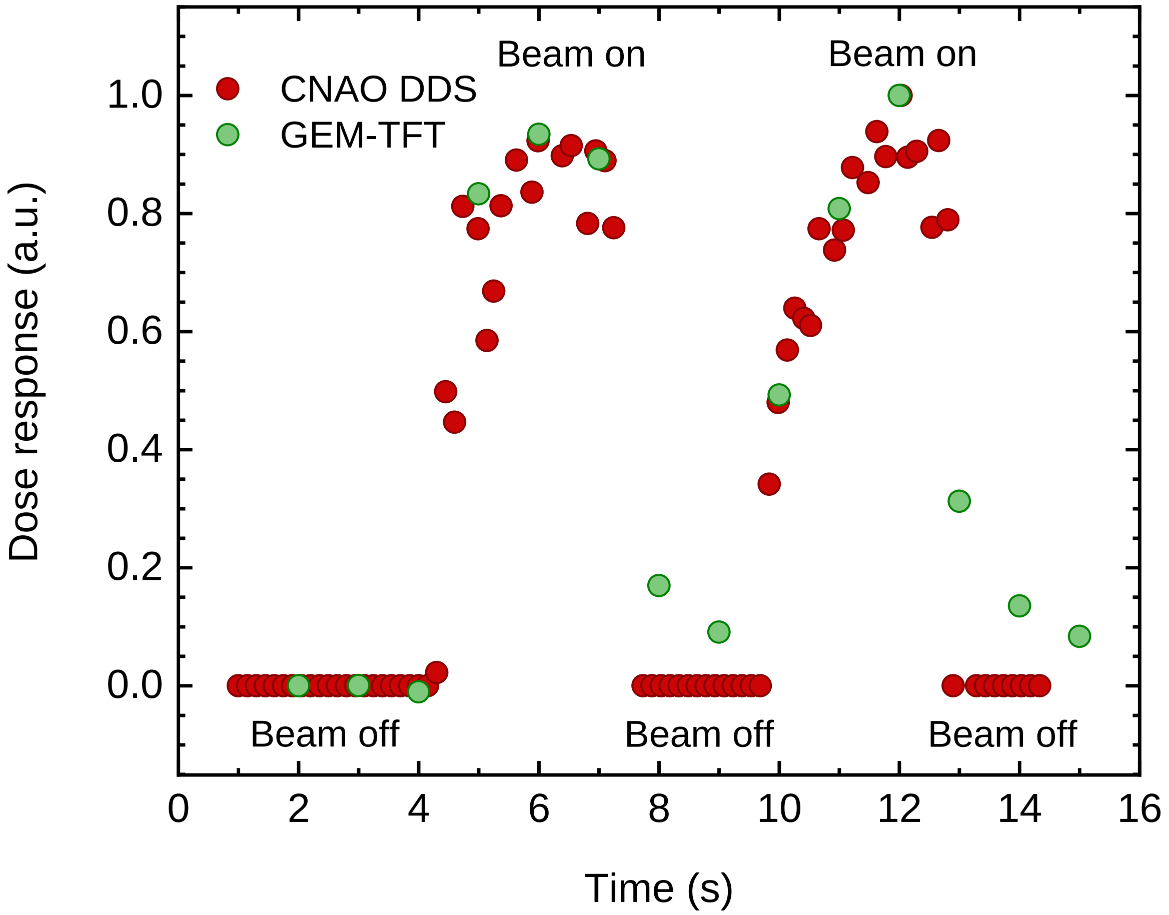

Supplementary Fig. 5 | Dose response vs. time for the GEM-TFTF detector and Dose Delivery System used at CNAO. Data normalized to the peak values. Subset of CNAO DDS data points shown (1 in every 3 points) to enhance clarity. The GEM-TFTF data points between spills remain non-zero due to 1 s frame rate, capturing beam ON and OFF.
